# Barriers in general practitioners’ dementia diagnostics among people with a migration background in Germany (BaDeMi) - study protocol for a cross-sectional survey

**DOI:** 10.1186/s12874-018-0580-0

**Published:** 2018-11-06

**Authors:** Judith Tillmann, Rieke Schnakenberg, Marie-Therese Puth, Klaus Weckbecker, Johannes Just, Eva Münster

**Affiliations:** 1 Institute of General Practice and Family Medicine, Medical Faculty of the University of Bonn, Sigmund-Freud-Str. 25, 53127 Bonn, Germany; 2 Department of Medical Biometry, Informatics and Epidemiology (IMBIE), University Hospital of Bonn, Sigmund-Freud-Str. 25, 53127 Bonn, Germany

**Keywords:** Dementia diagnostics, Family medicine, Migration background, Culture and health, Public health, Epidemiology, GP

## Abstract

**Background:**

Considering the targeted general practitioner-centred healthcare in Germany, general practitioners (GPs) are in the best possible position to increase awareness of all sorts of dementia, an age-related syndrome with rising relevance in the future. In Germany, a doubling of the number of cases from 1.55 million up to 3 million in 2050 is predicted. Diagnostics can be challenging, especially among patients with a migration background. Complicating factors include: Language-based diagnostic tools, cultural differences in handling the syndrome and its underlying diseases as well as a differing use of the healthcare system. Because of missing research in this field in Germany, the type, frequency and intensity of barriers as well as the way GPs cope with them is unknown. That is why it’s crucial to focus research on diagnostics in total and especially among this population group.

**Methods:**

A cross-sectional study among a random sample of 1000 general practitioners in Germany is conducted in October 2017. A self-administered standardized questionnaire was developed, evaluated and send to the GP practices. A response rate of 30% is expected with one reminder letter. Descriptive statistics as well as, depending on the results, multivariable analyses will be executed. Based on these results and the stated needs, a cluster-randomized intervention study will be constructed to improve healthcare.

**Discussion:**

This study is the first in Germany focusing on how dementia diagnostics in general practice is performed, what problems occur, especially because of a migration background of patients, and how GPs cope with them. Depending on the results, it should emphasize the necessity of dementia diagnostics to be adjusted to the needs of the rising amount of people with a migration background (22.5% in Germany, 2016) like concluded from international studies.

**Trial registration:**

German Clinical Trials Register: DRKS00012503, date of registration: 05.09.2017. Clinical register of the study coordination office of the University hospital of Bonn: ID530, date of registration: 05.09.2017.

## Background

Rising life expectancy in the course of demographic change causes a steep increase in the number of people with age-related diseases, notably dementia. A rise of actually 35.6 million patients with dementia worldwide (2010) up to 42 million patients till 2040 is predicted [1, 2]. In Germany, a country with 82.4 Million citizen, a doubling of the number of cases from 1.55 million up to 3 million in 2050 is predicted [3, 4], resulting in costs of 85 up to 142 billion € [2]. Above all, the growing number of people suffering from dementia is a huge public health challenge. Within the framework of action plans at national level, the World Health Organization (WHO) points out that awareness of dementia should be sharpened and early diagnosis should be supported [5]. General Practitioners (GPs) should generally be the first point of contact for people with health problems in Germany. This includes possible first symptoms of dementia like problems with short-term memory, concentration, orientation, mood or mental capacity [6]. Therefore GPs are in the best position to increase awareness of all types of dementia. Besides they can detect treatable reasons of the syndrome best to prevent permanent health impairment.

Because symptoms are difficult to distinguish from beginning forgetfulness due to aging and Mild Cognitive Impairment (MCI), diagnostics is challenging for many GPs, even among patients without a migration background. Moreover, limited consultation timeframes of GPs are likely to prevent precise diagnostics. Another aspect described in international literature is restricted knowledge of GPs about a good personal interaction with their dementia patients as well as existence of regional services to support people who are concerned [7–9].

Especially people with a migration background require particular attention because diagnostic tools of dementia are language-based, cross-cultural adaption is missing and the syndrome and underlying diseases are tabooed or handled differently in some cultures. Moreover a less frequent use of health services is reported in international as well as European studies [10–18]. The German Federal Statistical Office (Destatis) defines migration background as follows: Either a person itself or at least one parent is born without German nationality [19]. According to the Microcensus 2016, 22.5% of the population in Germany share this characteristic; a further increase in future is predicted [4]. Among this group, Turkey (15.1%), Poland (10.1%) and Russia (6.6%) represent the most common countries of origin [4].

Within European studies of Nielsen et al., two thirds of doctors describe diagnostics and classification of dementia among ethnic minorities as problematic [17]. There are further international hints that dementia is underdiagnosed among migrants [11, 16, 20]. In total, this topic has hardly been explored, especially in the European area, and requires particular attention [17, 21]. Hence this project is the first of its kind in Germany and represents a great step towards closing the research gap and improving healthcare of people suffering from dementia.

The following research question should be answered: Are there any barriers in dementia diagnostics in general practice, especially between GPs and patients with a migration background? And if so, what kind of barriers exist and how do GPs deal with them? In order to identify underlying causes, common methods and diagnostic tools used by GPs in the course of dementia diagnostics as well as their experiences with patients showing symptoms of dementia should be determined. Moreover it is of high relevance to gather what actions GPs suggest to remove barriers.

## Methods

### Study aim

The project “Barriers in general practitioners’ dementia diagnostics among people with a migration background” (BaDeMi) aims at improving dementia diagnostics in general practice through a reduction of barriers among doctors and patients. In the course of the cross-sectional study, experiences, potential barriers on the doctors’ and patients’ side as well as possibilities to improve dementia diagnostics should be detected. Special focus is laid on patients with a migration background since they could have special needs due to language barriers, lack of knowledge about the syndrome and the health system as well as cultural differences. Based on these results, information material will be developed and evaluated in the course of a cluster-randomized intervention study with the aim to reduce barriers and improve healthcare of people with dementia or related symptoms. All in all, health inequalities among people with and without a migration background regarding dementia care should be reduced.

### Design

**Fig. 1:**
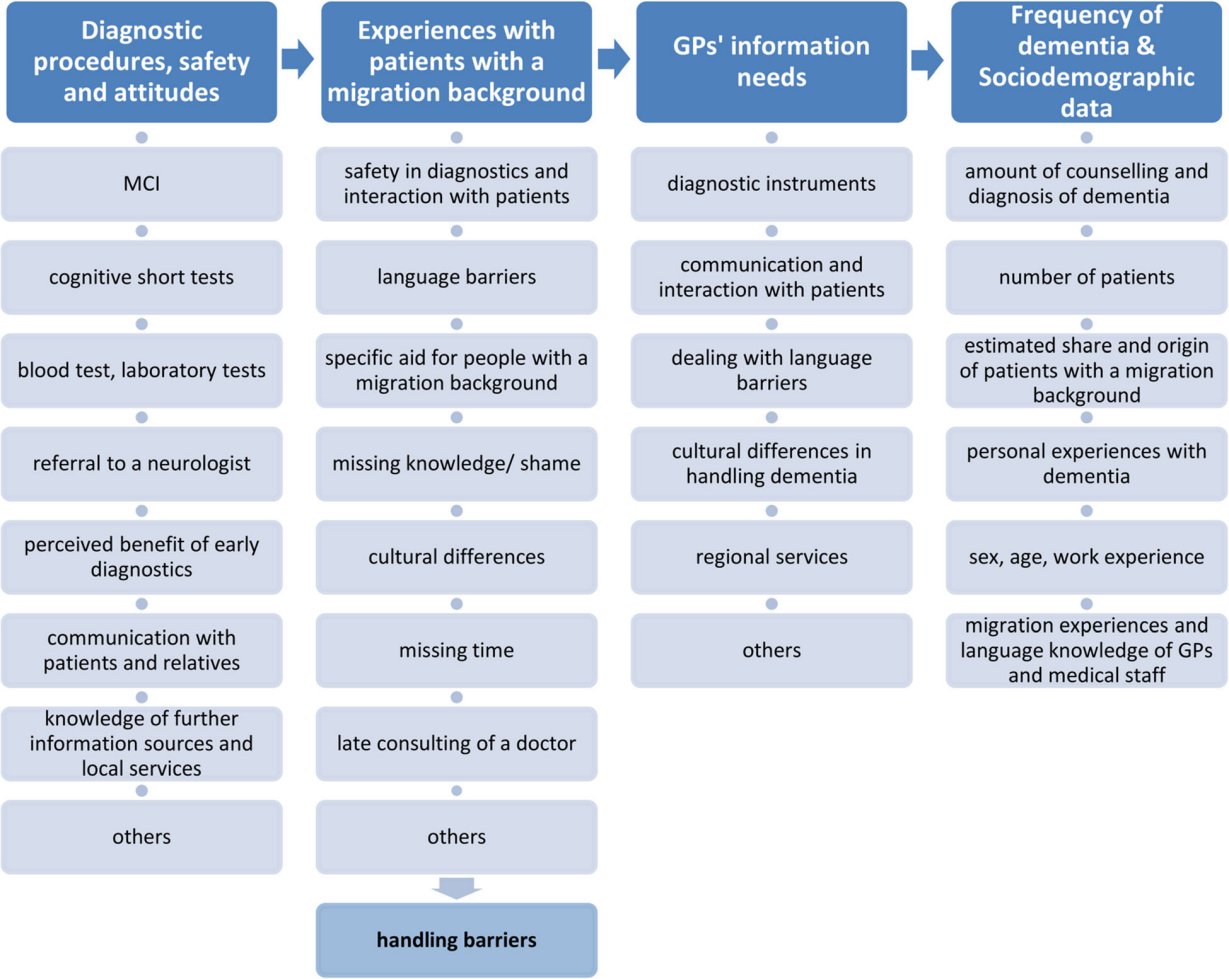
Main structure of the BaDeMi-questionnaire

The BaDeMi-project consists of two studies, a cross-sectional survey of general practitioners and a following cluster-randomized intervention study; the first one is central to this protocol: It is a cross-sectional survey among a random sample of 1,000 GPs in North Rhine-Westphalia, the most populous state of Germany (17.87 million inhabitants) [22]. The period of the whole project is set from May 2017 up to April 2019, the cross-sectional study will be conducted in the period of September 2017 to December 2017, while the survey takes place in October 2017. The standardized self-administered written survey includes questions about procedures of general practitioners used within the scope of dementia diagnostics as well as their experiences with patients with a migration background (Fig. 1). Finally, ways to improve diagnostics and support doctors in their practices are inquired. As response categories, five-point Likert-type scales with responses varying from strongly disagree to strongly agree as well as multiple choice response fields were used.

The questions were developed based on systematic literature research on the topic in medical databases (PubMed, Livivo) and Google Scholar. The most common problems in the diagnostic process and in dealing with patients with a migrant background described in the international literature were taken up as questions/answer options in the questionnaire. In addition, free text fields have been added to name additional aspects not yet described.

Eight questions about methods in diagnostics and physicians’ attitudes towards dementia at the beginning of the questionnaire are based on a survey developed by Australian researchers of Wicking Dementia Research & Education Centre (University of Tasmania) within the scope of collaboration [23]. Questions were translated using the method of back-translation by an English native speaker to ensure comparability.

### Setting and eligibility criteria

Primary target group of this study are general practitioners in North Rhine-Westphalia who practiced at the time of study execution. They have to be registered in the data of the Association of Statutory Health Insurance Physicians North-Rhine (Kassenärztliche Vereinigung Nordrhein (KVNO)) as a general practitioner on the 28th of July 2017 (*n* = 6,313). Doctors have to be approved in the KVNO in Germany to be allowed to treat patients with a statutory health insurance (87.7% of the population) [24]. These doctors are allowed to treat privately insured patients as well. This group of doctors contains GPs specialized on general medicine, practical physicians as well as internists. They work in practices, ambulatory healthcare centers (MVZ) or group practices. Pediatricians have been excluded because of lack of relevance for dementia research. All other doctors who are not labeled as GPs have been excluded (*n* = 15,007). Also, GPs who have exclusively private paying patients and are therefore not registered in the KVNO have not been included.

### Study endpoints

The aim of this cross-sectional study is the identification of barriers in dementia diagnostics in general practice among people with and without a migration background from physicians’ point of view. Using predefined categories and additional free text fields (Fig. 1), occurring problems should be defined, for example into problems caused by poor applicability of diagnostic instruments, missing knowledge of patients and/or GPs, communication or differences in culture. Second aim is the identification of established methods of dementia diagnostics in general practice as well as possible ways to support GPs in diagnostics of patients suffering from dementia.

### Sample size and recruitment

**Fig. 2:**
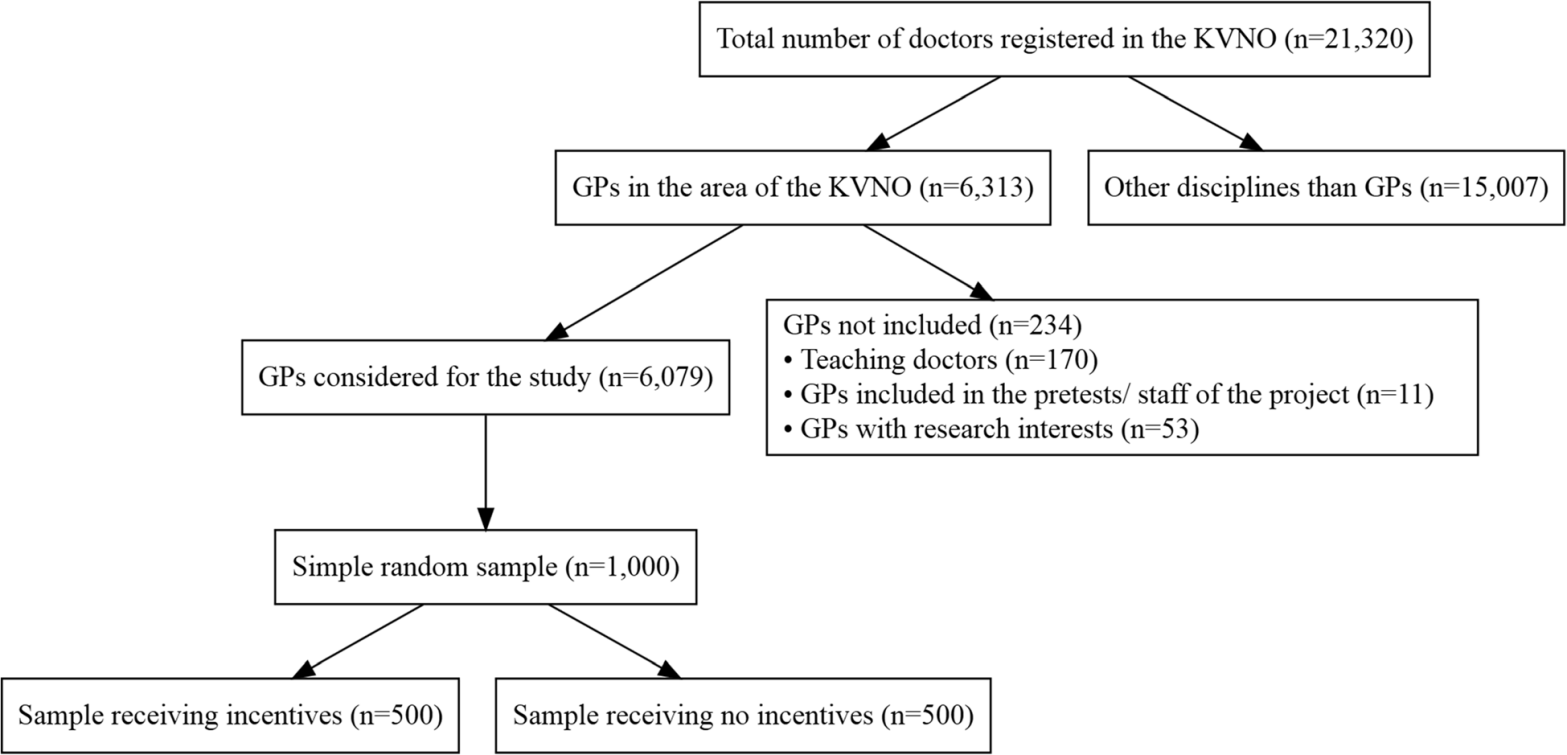
Procedure of selection of doctors enclosed in the cross-sectional study

Among 6313 general practitioners fitting the inclusion criteria, a random sample of 1000 doctors was enclosed in the study (Fig. 2). Before, GPs connected with the research institute like teaching doctors (*n* = 170), GPs working in the project (*n* = 2), being connected with research projects (*n* = 53) as well as GPs included in the pretest (*n* = 9) were excluded. This random sample has been contacted at the beginning of October 2017 with a letter that contains a covering letter, instructions, the pseudonymised questionnaire and an already addressed and postpaid envelope. They were asked to participate and send it back in the annexed envelope. Half of the study population (*n* = 500), randomly chosen, also received an incentive, typical sweets from the city of the study, in the envelope. Non-responders were contacted again in writing after two weeks and asked to participate with the questionnaire enclosed again.

### Pretesting

Before conducting the study, the questionnaire has been pretested among four general practitioners to ensure content validity and to identify possible sources of error. The GPs have not been contacted before. They received the questionnaire and a standardized evaluation sheet and were asked to evaluate the questionnaire following the categories on the evaluation sheet. They commented on comprehensibility and construction, relevance and quality, design and duration. The results of this pretest have been used to adapt the questionnaire to ensure suitability for daily use.

### Statistical analysis

The software IBM SPSS Statistics (Version 22) will be used for data analyses [25]. The questionnaires will be scanned by the data capture system TeleForm. All GPs who answer and resend the questionnaire will be included. Simple descriptive statistics including frequencies with 95% confidence intervals, medians, means and standard deviations will be used to describe the various parameters covering the evaluations of GPs of dementia and its diagnostics as well as sociodemographic determinants. Multivariable analyses, comprising multiple logistic regressions, will be conducted. Missing data will be coded and summarized. To reduce potential sources of bias, the study sample was randomly chosen and the questionnaire was standardised. Sociodemographic data like age, gender, migration background and other characteristics of GPs are gathered in the questionnaire and analyses will be adjusted for these characteristics. Moreover logistic regression analyses will be conducted stratified by gender and other characteristics of GPs to control the influence of this characteristic and avoid effect modification.

### Ethic approval, data management and funding

The BaDeMi study has been approved by the Ethics Committee of the Medical Faculty of the University of Bonn (no. 251/17). Moreover, it has been registered at the German Clinical Trials Register (DRKS) (no. DRKS00012503) and the clinical register of the study coordination office of the University Hospital of Bonn (ID530). Participation is optional for all doctors. By means of a covering letter, they get informed about the study, its importance and aims as well as benefits for healthcare. Person-identifying data, such as names and birthdays, have not been collected. Anonymity is ensured after receiving the questionnaires. All data will be stored under lock for at least ten years. The project is financially supported by the German Alzheimer Society (https://www.deutsche-alzheimer.de/). The study is conducted independently from the funder and competing interests. It is ensured that only staff members of the project get access to the collected data.

The results of this study will be published in articles in national and international medical journals and presented to healthcare professionals of the German Alzheimer Society. They will also be used to construct the following cluster-randomized intervention study.

## Discussion

This study aims at closing several gaps in dementia and migration research. Especially in Germany, only little has been done in general practice to improve care of people with dementia, especially with a migration background. It is unknown with what kind of barriers GPs are confronted, how frequently they occur and how they deal with them. In Germany, the overall proportion of people with a migration background is high. In the most populated state North Rhine-Westphalia, where the study is conducted, it is even higher (27,2%) [4]. Considering the rising amount of asylum seekers and refugees, who have not been in contact with the German healthcare system at all, it’s even more urgent to set adjustments on the doctor’s and patient’s side. Research in other parts of the world like Denmark and Australian is much more advanced. It already highlights the importance of adjusted medical approaches and dementia diagnostics in particular on the needs of the population with a migration background or so called “ethnic minorities” [11, 16, 17, 20, 21]. Nevertheless, setting the focus on GPs has not been done so far. Based on these results, information material will be developed and evaluated in a cluster-randomized intervention study at family medicine practices.

Results might differ between GPs because of different patient bases as well as the region of the practice and connected differing amount of patients with or without a migration background. GPs’ own migration background might also influence responses. Patients might rather consult GPs with the same migration background to feel comfortable and well understood. For this reason it is essential to gather these aspects in the survey and to consider them in the analyses.

Since study execution is limited to GPs registered in the Association of Statutory Health Insurance Physicians North-Rhine, results might differ from private doctors. Because 87.7% of the German population is insured in a statutory health insurance, that aspect won’t have large effects [24]. Furthermore there are other health professionals like neurologists and nursing staff that routinely interact with patients suffering from dementia, who should be researched in future regarding their experiences with people with a migration background. A response bias cannot be excluded as the responding GPs may be more interested in the topic. However, since characteristics of GPs, such as the age patterns, are quite similar to the average of GPs in Germany, the results may allow generalisation. Barriers and problems identified in this study may not be transferable to all migrant populations because of heterogeneous cultures, religions and views existing even within the barriers of a country. To improve diagnostics and therefore healthcare of people with dementia, the findings of this study will be used in the next step to develop information material for GPs as well as their patients.

## Abbreviations

Destatis: German Federal Statistical Office
DRKS: German Clinical Trials Register
GP: General Practitioner
MVZ: ambulatory healthcare center
WHO: World Health Organization
